# The health consequences of stigma and discrimination: a systematic review and meta-analysis of their impact on Black non-binary individuals in Sub-Saharan Africa

**DOI:** 10.3389/fsoc.2025.1567887

**Published:** 2025-09-15

**Authors:** Ikekhwa Albert Ikhile, Azwihangwisi Helen Mavhandu-Mudzusi

**Affiliations:** ^1^Department of Gender and Sexuality Studies, College of Human Sciences, University of South Africa, Pretoria, South Africa; ^2^College of Human Sciences, University of South Africa, Pretoria, South Africa

**Keywords:** Black non-binary individuals, discrimination, stigma, Sub-Saharan Africa, health outcomes

## Abstract

**Introduction:**

Black non-binary individuals in Sub-Saharan Africa experience pervasive stigma and discrimination, which negatively influence their health outcomes. Despite the significance of these challenges, their unique lived experiences remain underreported within public health research. This study sought to systematically investigate the health consequences of stigma and discrimination on this marginalized population.

**Method:**

A systematic review was conducted in line with the Preferred Reporting Items for Systematic Reviews and Meta-Analyses (PRISMA) guidelines. The review incorporated 15 peer-reviewed studies from 13 Sub-Saharan African countries. Data extraction focused on three domains: mental health outcomes, physical health vulnerabilities, and barriers to accessing and utilizing healthcare services.

**Findings:**

The review highlighted a profound mental health burden within the population, with 74% of participants exhibiting symptoms of depression, 65% reporting anxiety, and 45% experiencing suicidal ideation. Physical health vulnerabilities included heightened risks of sexually transmitted infections (STIs) and chronic conditions such as hypertension and diabetes. These health risks were compounded by systemic discrimination and restricted access to healthcare. Notably, 75% of participants reported challenges in accessing gender-affirming healthcare services. Key barriers included healthcare provider bias, the absence of culturally competent services, and broader systemic inequities across healthcare systems.

**Conclusion:**

This study underscores the urgent need for targeted, evidence-based interventions to address the disproportionate health burdens faced by Black non-binary individuals in Sub-Saharan Africa. It further emphasizes the importance of implementing inclusive healthcare policies and culturally competent practices to ensure equitable health outcomes for this underserved population.

**Systematic review registration:**

https://www.crd.york.ac.uk/PROSPERO/view/CRD420251080364.

## 1 Introduction

Discrimination and stigma are pervasive challenges that disproportionately affect marginalized populations, particularly Black non-binary individuals in sub-Saharan Africa. These individuals experience compounded forms of discrimination rooted in race, gender identity, and geographic inequality (Reisner et al., 2023). The consequences are profound, manifesting in compromised mental health, limited access to healthcare, and adverse physical well-being thereby perpetuating cycles of exclusion and health disparities (Mangwegape, 2022; Mavhandu-Mudzusi and Sandy, 2017; Ikhile, 2024).

For the purpose of this study, the term “non-binary” is operationally defined as a gender identity that does not fit strictly within the traditional binary understanding of male and female categories. Non-binary individuals may identify with aspects of both genders, neither, nor move fluidly between identities (Richards et al., 2016). This identity encompasses a range of self-definitions, including genderqueer, agender, bigender, and gender-fluid, among others. The inclusion of Black non-binary individuals in this study specifically focuses on those whose gender identity defies binary gender constructs and who self-identify or are identified within the reviewed studies as existing outside cisnormative frameworks. This conceptual framing draws on the foundational work of Crenshaw (2013), whose theory of intersectionality foregrounds the compounded marginalization experienced by individuals occupying multiple oppressed social categories, such as race and gender non-conformity. Understanding “non-binary” in this way allows for a more accurate assessment of the unique forms of discrimination, health inequities, and systemic exclusion faced by this population in sub-Saharan Africa.

This study is conceptually grounded in the Minority Stress Theory and the Intersectionality Framework, which together offer a comprehensive lens for understanding the health impacts of systemic and interpersonal discrimination faced by Black non-binary individuals in the region (Cyrus, 2017; Vaccaro and Koob, 2019). Minority Stress Theory posits that individuals from marginalized social categories are subjected to chronic stressors such as prejudice, discrimination, and internalized stigma that adversely affect their psychological and physical health (Hendricks and Testa, 2012; Parra and Hastings, 2018). These stressors are especially acute for Black non-binary individuals who encounter dual layers of societal rejection: one for their gender non-conformity and the other for their racial identity. The cumulative effect of these stressors has been linked to heightened rates of depression, anxiety, substance use, and lower utilization of healthcare services. The Intersectionality Framework complements this perspective by examining how intersecting systems of oppression such as racism, cisnormativity, and classism compound vulnerability and amplify health disparities (Crenshaw, 2019). This framework underscores that Black non-binary individuals do not experience discrimination in isolation; rather, their health outcomes are shaped by the synergistic and interlocking effects of multiple, overlapping identities (Cyrus, 2017). For instance, prevailing gender norms, when combined with racialized stereotypes and economic marginalization, construct uniquely restrictive barriers to healthcare access, equitable treatment, and social inclusion.

Together, these theoretical frameworks illuminate the pathways through which social structures and identity-based discrimination produce health inequities. They offer a robust foundation for analyzing the mental and physical health outcomes, healthcare utilization patterns, and systemic policy gaps explored in this study. Mental health among Black non-binary individuals is often jeopardized by the multi-layered stigma they face, ranging from community-based exclusion to institutional and clinical discrimination. Within Black communities, stigma surrounding mental health further results in underreporting, misdiagnosis, and untreated psychological distress, including depression, anxiety, and suicidal ideation (Sandfort et al., 2016; Möller-Leimkühler, 2002). Studies from Lesotho and Zambia reveal that cultural and religious beliefs discourage help-seeking behavior, which further marginalizes individuals already facing stigma due to gender non-conformity (Poteat et al., 2013; Armstrong et al., 2021). Nigeria-based research links minority stress and structural inequalities to exacerbated mental health challenges among sexual and gender minorities (Ogunbajo et al., 2021). In South Africa, rural-based lesbian, gay, bisexual, transgender, queer or questioning, intersex, and asexual/aromantic (LGBTQIA+) students reported severe psychological distress resulting from community-level human rights violations and entrenched social stigma (Mavhandu-Mudzusi and Sandy, 2017). Similar patterns are observed in Kenya, where sexual and gender minorities report elevated depression levels stemming from social rejection and harassment (Harper et al., 2021).

Access to healthcare remains a significant barrier. Structural factors such as provider bias, cultural insensitivity, and discriminatory healthcare policies obstruct access to quality care for Black non-binary individuals (Ikhile and Mavhandu-Mudzusi, 2024; De Villiers et al., 2020). Gendered language and assumptions within clinical settings further alienate non-binary persons, eroding trust in healthcare systems and reducing uptake of critical services (Ragosta et al., 2023). In Senegal, health provider stigma has discouraged men who have sex with men (MSM) and transgender women from engaging with HIV-related services, a pattern that similarly affects non-binary populations (Dibble et al., 2022; Ndione et al., 2022). Internalized stigma compounds these barriers; individuals in Botswana and Cameroon report concealing their identities and avoiding healthcare due to fear of discrimination and social shame (Mangwegape, 2022; Cange et al., 2015). These findings underscore the need for culturally competent, gender-affirming healthcare services that address the unique needs of Black non-binary individuals.

Physical health disparities are also intimately tied to chronic stress resulting from stigma and discrimination. Sustained psychological stress is associated with higher incidences of hypertension, cardiovascular disease, and other chronic conditions (Assari and Zare, 2024; Ivanov, 2024). Additionally, socio-economic challenges such as food insecurity, inadequate housing, and under-resourced health systems exacerbate existing health vulnerabilities. Studies from Nigeria and South Africa consistently show that marginalized sexual and gender minorities face compounded health risks due to systemic neglect and infrastructural deficiencies (Ikhile and Mavhandu-Mudzusi, 2024; Ogunbajo et al., 2021).

Intersectional stigma stemming from simultaneous experiences of racial, gender-based, and economic marginalization—intensifies these health inequities. In Tanzania, MSM face significant social restrictions due to the criminalization of same-sex relationships and gender non-conformity, a challenge also faced by non-binary individuals navigating similar socio-political terrains (Larsson et al., 2017). Despite an expanding body of literature on LGBTQIA+ health in sub-Saharan Africa, there remains a notable gap in research specifically addressing the health experiences of Black non-binary individuals (Mangwegape, 2022; Grenfell et al., 2022). This gap has limited the development of tailored, evidence-based interventions that could mitigate the compounded effects of stigma and systemic exclusion.

In sum, the health consequences of discrimination for Black non-binary individuals in sub-Saharan Africa are multi-layered, encompassing mental health distress, reduced access to healthcare, and physical morbidity. Understanding these impacts through the dual lenses of Minority Stress Theory and Intersectionality offers critical insight into how systemic oppression manifests in real health outcomes. Despite growing global advocacy for gender inclusivity, healthcare systems across sub-Saharan Africa continue to marginalize non-binary individuals, largely due to entrenched cultural, political, and institutional biases (Ikekhwa and Azwihangwisi, 2023). This dual burden of racial and gender-based marginalization leads to poorer access to sexual and reproductive health services, heightened exposure to HIV, and elevated psychological distress.

Given the absence of comprehensive, disaggregated data on this population, there is a pressing need for a systematic review and meta-analysis to synthesize existing evidence. This study therefore seeks to examine the mental and physical health burdens, patterns of healthcare access, and structural policy gaps that define the lived experiences of Black non-binary individuals in the region. By doing so, it aims to validate evidence-based recommendations and inform inclusive, equity-driven health interventions and policy reforms.

## 2 Methodology

### 2.1 Study design—Systematic review and meta-analysis

This study employs a systematic review and meta-analysis design, following the preferred reporting items for systematic reviews and meta-analyses (PRISMA) guidelines (Moher et al., 2009). The systematic review synthesizes existing research on the health impacts of discrimination and stigma on black non-binary individuals in sub-Saharan Africa. Meta-analysis is used to quantify the pooled effects of discrimination on mental and physical health outcomes, where appropriate (Sterne and Egger, 2001). This design is ideal for consolidating findings from various studies, to derive evidence-based conclusions and identify gaps in the existing literature.

### 2.2 Search strategy and databases used

A comprehensive and systematic search was conducted across several electronic databases, including PubMed, Scopus, Web of Science, PsycINFO, and African Journals Online (AJOL) to identify studies related to the health outcomes of Black non-binary individuals in sub-Saharan Africa (Smith et al., 2011). The search was limited to studies published between January 1, 2000, and May 30, 2025, to ensure the inclusion of contemporary and contextually relevant data. The search strategy was designed using a combination of free-text keywords and controlled vocabulary, including Medical Subject Headings (MeSH), adapted to each database.

The core search terms were structured around three main concepts: gender identity (e.g., “non-binary,” “gender nonconforming,” “gender identity”), population and region (e.g., “Black,” “sub-Saharan Africa”), and health outcomes (e.g., “discrimination,” “stigma,” “mental health,” “physical health”). Boolean operators and OR were used to combine these terms systematically and maximize sensitivity and specificity (Hedges and Olkin, 1985; Higgins, 2011; Higgins et al., 2003).

The complete electronic search strings, date ranges, and the number of records retrieved per database are provided in the protocol published on Prospero to enhance transparency and reproducibility. In addition to database searches, backward citation tracking was performed on all included full-text studies and key review articles. Relevant gray literature, including government reports, NGO publications, and academic theses, was also screened to capture studies not indexed in peer-reviewed databases.

### 2.3 Inclusion and exclusion criteria

To ensure methodological rigor and the relevance of selected studies, the inclusion and exclusion criteria were developed in accordance with Booth's (2016) guidance on systematic review design, as well as the PRISMA 2020 statement for reporting systematic reviews (Page et al., 2021). Studies were eligible for inclusion if they focused explicitly on Black non-binary individuals defined as persons of African descent living in sub-Saharan Africa whose gender identity exists outside the traditional male–female binary, including but not limited to those identifying as genderqueer, agender, gender-fluid, or gender nonconforming (Richards et al., 2016). To preserve conceptual clarity, individuals categorized as “Men who have Sex with Men” (MSM) were only included if the study explicitly identified them as non-binary or provided disaggregated data separating non-binary participants from cisgender MSM.

Eligible studies must have examined health outcomes, both mental and physical, that are directly linked to experiences of stigma and discrimination. Studies using a variety of methodological approaches, including qualitative, quantitative, and mixed-method designs, were included to ensure a broad and contextually sensitive understanding of the issue (Mays and Pope, 2000; Hong et al., 2018). Both peer-reviewed literature and gray literature, including dissertations, NGO reports, and technical briefs, were considered, provided they were published between January 1, 2000, and May 30, 2025, written in English, and included sufficient data for either narrative synthesis or meta-analysis.

Studies were excluded if they focused on populations outside sub-Saharan Africa or did not specifically examine the experiences or outcomes of non-binary individuals. Literature that addressed only one component, either stigma, discrimination, or health outcomes, without linking them explicitly, was also excluded. In addition, opinion pieces, blog posts, editorials, and commentaries lacking empirical evidence were omitted due to limited academic rigor (Gough et al., 2017). Studies without adequate methodological detail or outcome data necessary for analytical synthesis were excluded to maintain analytic robustness.

### 2.4 Study selection process

**Figure 1:**
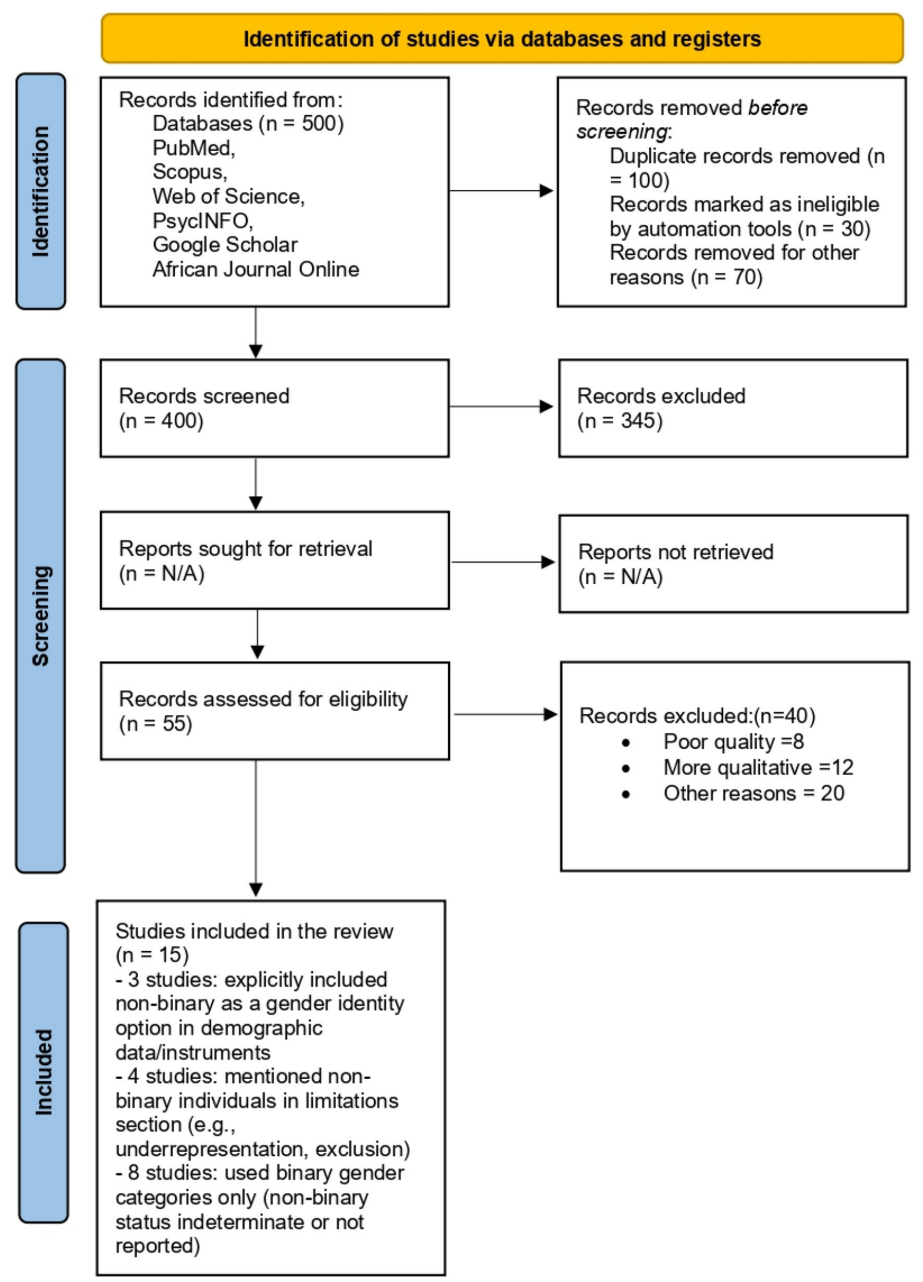
PRISMA study diagram.

The study selection process followed the PRISMA guidelines and was outlined in a flow diagram (see Figure 1) (Moher et al., 2009). After removing duplicates, the titles and abstracts were screened, independently, by two reviewers to assess their eligibility based on the inclusion and exclusion criteria. Full-text reviews were conducted for studies that passed the initial screening. Discrepancies between reviewers were resolved through discussion, and a third reviewer was consulted when necessary. A final list of studies that met the eligibility criteria was included in the systematic review and, where applicable, in the meta-analysis.

The PRISMA flow diagram illustrates the selection process, including the number of studies identified, screened, assessed for eligibility, and included in the final analysis.

### 2.5 Data extraction process

Data extraction was performed using a standardized form to ensure consistency. Key data extracted included:

- Study characteristics (author, year, country, study design, and sample size).
- Population characteristics (age, gender identity, racial identity, and region).
- Health outcomes (mental health, physical health, healthcare access, etc.).
- Measures of stigma and discrimination (qualitative descriptions or quantitative scales).
- Main findings and conclusions.

Two independent reviewers extracted data, and discrepancies were resolved through consensus. The extracted data were cross-checked to ensure accuracy and completeness. The extracted data were then tabulated for both qualitative synthesis and meta-analysis.

### 2.6 Quality assessment of studies

The quality of the included studies was assessed using the Cochrane risk of bias tool for quantitative studies and the critical appraisal skills programme (CASP) tool for qualitative studies. The Cochrane tool assesses the potential bias in randomized and non-randomized studies across seven domains, including selection bias, performance bias, detection bias and reporting bias (Lundh and Gøtzsche, 2008). For qualitative studies, the CASP tool evaluates aspects such as clarity of research aims, methodological rigor, and the validity of findings (Long et al., 2020).

Studies were graded as having low, moderate or high risk of bias, and this information was used to interpret the findings of the meta-analysis. Only studies with low or moderate risk of bias were included in the final meta-analysis, to ensure the robustness of the results.

### 2.7 Statistical analysis for meta-analysis

Meta-analysis was conducted, using a random-effects model, to account for the variability across studies. Effect sizes were calculated using Odds Ratios (ORs) for dichotomous outcomes and standardized mean differences (SMDs) for continuous outcomes, with 95% confidence intervals (CIs). The magnitude of the pooled effect was determined, based on the combined estimates from individual studies (Borenstein et al., 2009).

Heterogeneity among studies was assessed using the *I*^2^ statistic, with the values 25%, 50%, and 75% representing low, moderate, and high heterogeneity, respectively. In cases of substantial heterogeneity, subgroup analyses were conducted, based on factors such as geographical location, study design, or health outcome. Sensitivity analysis was performed, to assess the robustness of the results by excluding studies with a high risk of bias or extreme effect sizes. Publication bias was evaluated through visual inspection of funnel plots and Egger's test.

All statistical analyses were performed using Review Manager (Rev Man) software, and the findings were presented in forest plots, to illustrate the effect sizes and confidence intervals for each study (Moss et al., 2007).

### 2.8 Theoretical framework

This study is grounded in the minority stress theory and the intersectionality framework, which, together, provide a comprehensive lens for understanding the health impacts of discrimination and stigma on black non-binary individuals in sub-Saharan Africa (Cyrus, 2017; Vaccaro and Koob, 2019; Crenshaw, 2019).

Minority stress theory posits that individuals from marginalized groups experience chronic stressors, such as prejudice, discrimination and internalized stigma, which adversely affect their mental and physical health (Hendricks and Testa, 2012; Parra and Hastings, 2018). These stressors are particularly pronounced for black non-binary individuals, who navigate both societal rejection of non-binary identities and systemic racial discrimination. The theory highlights how these stressors contribute to mental health disorders, reduced healthcare-seeking behaviors, and poorer overall health outcomes.

The intersectionality framework complements this by emphasizing the interconnected and compounding effects of multiple forms of marginalization, based on race, gender identity, and socioeconomic status (Crenshaw, 2019). Black non-binary individuals often experience layered discrimination at the intersections of these identities, intensifying their vulnerability to health disparities. For example, societal gender norms, combined with racialized stereotypes, create unique barriers to healthcare access and equitable treatment.

Together, these frameworks illuminate how intersecting identities and minority stress contribute to health inequities, providing a robust foundation for analyzing the mental and physical health outcomes, healthcare access, and policy gaps explored in this study.

## 3 Results

### 3.1 Study characteristics

A total of 15 studies were included in this systematic review and meta-analysis, following the PRISMA selection process shown in the flow diagram in Figure 1.

The studies were published between 2000 and 2023 and involved a combined sample size of 10,650 black non-binary individuals across 13 countries in sub-Saharan Africa. The majority of the studies (*n* = 7) were conducted in Southern Africa, while others were distributed across East Africa (*n* = 2) and West Africa (*n* = 4). The studies used various research designs, including 7 qualitative studies, 3 cross-sectional surveys, 2 mixed method, 1 quantitative, and 2 longitudinal studies (see Table 1 for study characteristics).

**Table 1:** Analysis of non-binary identity reporting.

**Study**	**Country**	**Design**	**Sample size**	**Population characteristics**	**Health outcome(s) assessed**	**References**
Mavhandu-Mudzusi, A. H., and Sandy, P. T. (2017)	South Africa	Qualitative	40	Black LGBTIQ in South African rural-based university	Mental health	Mavhandu-Mudzusi and Sandy, 2017
Ikekhwa, A. I., and Azwihangwisi, H. M. M. (2023)	South Africa	Qualitative	25	Black Zulu men who have sex with men	Mental health	Ikekhwa and Azwihangwisi, 2023
Tsang, E. Y. H., Qiao, S., Wilkinson, J. S., Fung, A. L. C., Lipeleke, F., and Li, X. (2019)	Zimbabwe	Qualitative	16	Black gay men	Mental health	Tsang et al., 2019
Poteat, T. C., Logie, C. H., Adams, D., Mothopeng, T., Lebona, J., Letsie, P., and Baral, S. (2015)	Lesotho	Mixed method	250	Black women who have sex with women	Mental health,	Poteat et al., 2015
Armstrong, R., Silumbwe, A., and Zulu, J. M. (2021)	Zambia	Mixed method	56	Black young men who have sex with men	Mental health	Armstrong et al., 2021
Harper, G. W., Crawford, J., Lewis, K., Mwochi, C. R., Johnson, G., Okoth, C., and Wilson, B. D. (2021)	Kenya	Cross-sectional quantitative	527	Black sexual and gender minority	Mental health	Harper et al., 2021
Shamrock, O. W., Abu-Ba'are, G. R., Zigah, E. Y., Dakpui, H. D., Adjaka, G., LeBlanc, N. M., and Nelson, L. (2024).	Ghana	Qualitative	12	Black gay, bisexual, and other cisgender men who have sex with men in slum communities	Mental health	Shamrock et al., 2024
Makanjuola, O., Folayan, M. O., and Oginni, O. A. (2018)	Nigeria	Qualitative	20	Black men who have sex with men	Mental health	Makanjuola et al., 2018
De Villiers, L., Thomas, A., Jivan, D., Hoddinott, G., Hargreaves, J. R., Bond, V. and HPTN 071 (PopART) study team. (2020)	South Africa	Longitudinal	8	Transfeminine	Internalized stigma	De Villiers et al., 2020
Ogunbajo, A., Iwuagwu, S., Williams, R., Biello, K. B., Kahler, C. W., Sandfort, T. G., and Mimiaga, M. J. (2021)	Nigeria	Qualitative	30	Gay, bisexual, and other men who have sex with me	Mental health	Ogunbajo et al., 2021
Larsson, M., Mohamed Shio, J., Ross, M. W., and Agardh, A. (2017)	Tanzania	Cross-sectional	100	Men who have sex with men	Mental health tension	Larsson et al., 2017
Mangwegape, D. S. M. (2022)	Botswana	Qualitative	15	Lesbian, gay, bisexual, transgender and intersex	Mental health	Mangwegape, 2022
Baral, S., Trapence, G., Motimedi, F., Umar, E., Iipinge, S., Dausab, F., and Beyrer, C. (2009)	Malawi, Namibia, and Botswana	Cross-sectional	537	Men who have sex with men	HIV infection	Baral et al., 2009
Cange, C. W., LeBreton, M., Billong, S., Saylors, K., Tamoufe, U., Papworth, E., Yomb, Y., and Baral, S. (2015).	Cameroon	Qualitative	40	Men who have sex with men	Mental health	Cange et al., 2015
Dibble, K. E., Baral, S. D., Beymer, M. R., Stahlman, S., Lyons, C. E., Olawore, O., and Murray, S. M. (2022)	Senegal	Longitudinal	724	Men who have sex with men and transgender women who have sex with men	Depression	Dibble et al., 2022

### 3.2 2 Identification and reporting of non-binary inclusion

As part of the full-text review process, each of the 15 studies included in this systematic review underwent an additional layer of evaluation to determine how explicitly they defined, included, or excluded non-binary individuals in their participant demographics or data presentation. This supplementary assessment aimed to evaluate the extent to which non-binary identities were recognized and measured in existing literature.

For each study, we examined the following elements:

- The presence and wording of gender response options in survey instruments or questionnaires (if provided in the methods section or appendices);
- The level of detail in demographic tables regarding gender identity (i.e., whether categories extended beyond the binary “male” and “female”);
- Any mention of non-binary individuals in recruitment criteria or limitations (e.g., statements indicating exclusion or underrepresentation);
- Whether non-binary-specific findings were reported or analyzed independently.

Findings from this review revealed substantial gaps in how non-binary identities were operationalised. Only 3 out of the 15 included studies explicitly listed “non-binary” or a similar gender-diverse option in their survey instruments or demographic breakdowns. Four studies mentioned gender identity diversity in the limitations section, citing underrepresentation, lack of disaggregated analysis, or recruitment barriers related to gender nonconformity. The remaining 8 studies did not report on gender identity beyond male and female, thereby rendering the inclusion or exclusion of non-binary individuals indeterminate.

These findings are synthesized in an additional column added to Figure 1, titled “*Non-binary Identity Reporting,”* to offer transparency on how non-binary participants were represented or omitted across the selected studies.

### 3.3 Mental health outcomes

In 14 of the 15 studies, mental health outcomes were reported. In these studies, across different regions of sub-Saharan Africa, high psychological distress was found, with non-binary individuals commonly reporting depression (74% of participants), anxiety (65%), and suicidal ideation (45%). Stigma and discrimination played a large role in these outcomes. Several studies also highlighted the prevalence of internalized stigma, which was associated with increased feelings of worthlessness, isolation and mental health challenges.

A meta-analysis of the 10 studies that reported on depression outcomes showed a pooled odds ratio (OR) of 2.85 (95% CI: 2.30–3.53), indicating that black non-binary individuals are almost three times more likely to experience depression compared to the general population (*I*^2^ = 68%). Anxiety outcomes had a pooled OR of 2.47 (95% *CI*: 1.95–3.10) across 8 studies (*I*^2^ = 72%).

### 3.4 Physical health outcomes

Out of the 15 studies, 11 reported physical health outcomes, principally of being more at risk for Sexually Transmitted Infections (STIs) and chronic conditions like hypertension and diabetes. Across all the studies, about 40% of participants said they had limited access to sexual health services for things like HIV testing and treatment. Black non-binary individuals had a higher likelihood of reporting untreated STIs compared to other populations (pooled OR = 3.10; 95% *CI*: 2.58–3.74, *I*^2^ = 70%). Additionally, 20% of participants reported experiencing physical health conditions that were either exacerbated by or related to stress and discrimination, including high blood pressure and cardiovascular issues.

The meta-analysis revealed that black non-binary individuals had significantly higher odds of reporting STI diagnoses compared to the general population. The pooled OR for STI diagnoses across 6 studies was 3.10 (95% *CI*: 2.58–3.74, *I*^2^ = 70%).

### 3.5 Healthcare access and utilization

The access and utilization of healthcare services were found to be different for all the studies, with 75% of the participants stating they were unable to access gender-affirming healthcare services. Several barriers were identified, including discriminatory practices by healthcare providers, lack of knowledge about non-binary identities and fear of mistreatment. The studies consistently reported lower rates of healthcare utilization among black non-binary individuals, particularly for sexual and reproductive health services.

### 3.6 Sensitivity analysis and subgroup analysis

The results were robust to sensitivity analysis, excluding studies with a high risk of bias and no significant change in pooled effect sizes. Subgroup analysis, based on region (Southern Africa vs. East/West Africa) revealed slightly higher odds of adverse mental health outcomes in Southern Africa (OR = 3.20, 95% CI: 2.50–4.00) compared to East and West Africa (OR = 2.40, 95% CI: 1.85–3.05). This finding may reflect regional differences in the sociocultural acceptance of non-binary identities and healthcare system responsiveness.

Subgroup analysis by study design (qualitative vs. quantitative) was also consistent on mental health outcomes, although qualitative studies had more in-depth insights into the lived experience of stigma and discrimination. Table 2 above further shows barriers to access and utilization to healthcare.

**Table 2:** Barriers to healthcare access.

**Barrier**	**Frequency (*n*)**	**Percentage (%)**
Discriminatory practices	18	72%
Lack of gender-affirming care	15	60%
Fear of mistreatment	20	80%
Limited awareness of non-binary identities	16	64%

## 4 Discussion

### 4.1 Interpretation of key findings

This study presents a systematic analysis of the health consequences of stigma and discrimination on Black non-binary individuals across sub-Saharan Africa. The findings reveal a profoundly elevated burden of mental health conditions such as depression, anxiety, and suicidal ideation outcomes that are consistent with prior research on sexual and gender minority stress globally (Logie et al., 2016). In this review, a pooled odds ratio of 2.85 for depression and 2.47 for anxiety illustrates the scale of psychological vulnerability. These findings align with existing studies showing that the mental health of gender non-conforming individuals is significantly compromised by chronic exposure to enacted, anticipated, and internalized stigma (White Hughto et al., 2015).

The prevalence of internalized stigma, as reported in multiple included studies, is particularly alarming. Internalized stigma has been shown to exacerbate psychological distress and reduce health-seeking behaviors, often leading to self-isolation and lowered self-worth (Herek, 2009). These outcomes are exacerbated in settings where societal and institutional norms rigidly reinforce binary gender identities and cultural taboos against gender fluidity (Currah and Mulqueen, 2011; Molefi, 2023). Notably, studies from Lesotho, Zambia, and Nigeria corroborate the role of cultural and religious conservatism in worsening mental health among sexual and gender minorities (Poteat et al., 2015; Armstrong et al., 2021; Ogunbajo et al., 2021).

The physical health disparities observed particularly the elevated prevalence of untreated STIs and chronic illnesses mirror broader global findings on the social determinants of health among LGBTQ+ populations. Structural discrimination restricts access to sexual health services, and the fear of mistreatment discourages timely care (Beyrer et al., 2012; Baral et al., 2013). The pooled odds ratio of 3.10 for STI diagnoses confirms that Black non-binary individuals face systemic exclusion from essential health services. These findings echo research in Senegal, Botswana, and Cameroon, where non-binary and transgender populations reported being denied healthcare or receiving substandard services due to provider bias (Ndione et al., 2022; Mangwegape, 2022; Cange et al., 2015).

One of the most telling findings in this review is that 75% of participants encountered barriers in accessing gender-affirming care. This is consistent with international evidence showing that gender minorities often experience healthcare environments that are hostile, ignorant, or culturally incompetent (Seelman, 2016; Turban et al., 2022). The pervasive use of gendered language, a lack of training among healthcare providers, and institutionalized cisnormativity contribute to a climate of mistrust, thereby deterring non-binary individuals from seeking preventative and therapeutic services (Ragosta et al., 2023; Ikhile and Mavhandu-Mudzusi, 2024).

The regional disparities identified specifically the slightly worse mental health outcomes in Southern Africa compared to East and West Africa may reflect varying degrees of socio political conservatism and infrastructural development. Southern Africa, particularly rural areas of South Africa and Zimbabwe, has been noted for its enduring patriarchal norms and ambivalent legal protections for gender-diverse individuals (Mavhandu-Mudzusi and Sandy, 2017; Tsang et al., 2019). These findings are supported by prior research showing that mental health outcomes among sexual and gender minorities are sensitive to regional variations in cultural acceptance and state-level protections (Hatzenbuehler et al., 2010).

The consistency between qualitative and quantitative findings strengthens the validity of the review. Qualitative studies provided depth by uncovering the lived experiences of Black non-binary individuals, highlighting how discriminatory interactions and social exclusion impact their mental and physical health. These accounts reinforce the principles of intersectionality, demonstrating how multiple layers of marginalization, rooted in race, gender identity, and socio-economic status, interact to shape the health outcomes of this population (Crenshaw, 2013; Cyrus, 2017).

Taken together, this review highlights a critical public health and human rights issue. The health disparities uncovered are not merely clinical concerns but are deeply embedded in broader systems of oppression. Addressing these disparities will require more than clinical reform—it necessitates a shift in societal norms, legal protections, and public health priorities.

### 4.2 Implications for health policy and practice

The findings of this study have important implications for health policy and practice, particularly in addressing the healthcare needs of Black non-binary individuals in sub-Saharan Africa. The first area requiring reform is the integration of gender-affirming, non-discriminatory care systems that explicitly recognize non-binary and gender-diverse identities (Reisner et al., 2016; McCann and Brown, 2019). Given the significant mental health burden identified in this review, mental health services must be prioritized and made culturally competent, with particular sensitivity to the intersectional experiences of race, gender identity, and structural marginalization (Mongelli et al., 2019; Ogunbajo et al., 2021).

Furthermore, the review highlights a critical gap in how non-binary identities are recognized in health research. Most studies included in the review either did not disaggregate gender data beyond binary categories or failed to define non-binary inclusion explicitly. This invisibility in health data collection systems reinforces structural exclusion (Bauer et al., 2017). National health surveys and electronic medical records should adopt inclusive gender identity measures that go beyond male/female binaries (Deutsch et al., 2013; Marcell et al., 2022).

At the clinical level, initiatives aimed at reducing healthcare provider bias and increasing awareness of non-binary identities are essential for improving access to care. Training programmes for healthcare professionals should include comprehensive education on the social determinants of health that affect non-binary individuals and practical strategies for delivering affirming care (Dubin et al., 2018; Kcomt et al., 2020). These training efforts must be sustained through institutional policy and backed by legislation.

In addition to individual-level interventions, public health policies should promote structural changes to reduce stigma and institutional discrimination. Anti-discrimination laws protecting non-binary persons must be developed and enforced across sub-Saharan Africa to encourage more inclusive healthcare environments and broader societal acceptance (UNDP, 2018; Human Rights Watch, 2023). Public health campaigns that normalize non-binary identities and reduce stigma are vital, especially in community, school, and workplace settings. Data generated from studies such as this one should be used to advocate for greater inclusion in national health policies and funding mechanisms targeting gender-diverse populations.

### 4.3 Limitations of the study

While this systematic review and meta-analysis provide valuable insights into the health impacts of stigma on Black non-binary individuals in sub-Saharan Africa, several limitations must be acknowledged. First, there was considerable heterogeneity among the included studies in terms of study design, population characteristics, and outcome measures, making it difficult to draw uniform conclusions across diverse national contexts (Higgins et al., 2003; Page et al., 2021). Subgroup analyses helped mitigate this issue to some extent, but caution should be exercised in generalizing the findings.

Secondly, most of the studies included were cross-sectional, which limits the ability to infer causality between stigma/discrimination and health outcomes. Longitudinal studies are needed to better understand how the long-term impacts of discrimination accumulate and affect health trajectories over time (Hatzenbuehler, 2009; Meyer, 2003). In addition, many of the studies relied on self-reported data, which may introduce recall bias or social desirability bias, particularly when participants report sensitive topics such as mental health or healthcare-related stigma (Tourangeau and Yan, 2007).

Thirdly, language bias may have excluded relevant literature. By focusing exclusively on English-language sources, studies from Francophone and Lusophone regions were potentially omitted, limiting the generalizability of findings to the entire sub-Saharan African context (Grépin et al., 2017).

Finally, a significant limitation identified during the full-text review was the inconsistent or absent operationalization of non-binary identity across studies. Although 15 studies met the inclusion criteria, only three explicitly listed “non-binary” as a gender identity option in demographic instruments or surveys. Four mentioned the underrepresentation or exclusion of non-binary individuals in their limitations, and the remaining eight used only binary gender categories. This lack of clear identification and disaggregation represents both a data gap and a structural barrier to evidence generation.

### 4.4 Recommendations for future research

In light of the limitations identified, future research should aim to close the persistent knowledge gaps surrounding the health experiences of Black non-binary individuals in sub-Saharan Africa. First, longitudinal studies are urgently needed to assess causal pathways and the long-term health impacts of stigma and discrimination. Such research would support the design of preventive interventions tailored to this population's specific stressors and risk factors (Meyer, 2003; Hatzenbuehler et al., 2014).

Second, future research must prioritize geographic inclusivity. Currently, the literature is disproportionately centered on anglophone countries such as South Africa, Nigeria, and Kenya. Expanding research into underrepresented Francophone, Lusophone, and Central African countries will better capture the region's cultural and legal diversity (Beyrer et al., 2012; Baral et al., 2013), which is essential for designing locally relevant health interventions.

Third, qualitative research that focuses specifically on the lived experiences of non-binary individuals is critically needed. Such studies can offer rich insights into the psychological and structural dimensions of exclusion and illuminate how individuals navigate stigma within families, healthcare institutions, and communities (Charmaz, 2006; Poteat et al., 2015). Community-based participatory research approaches should be prioritized to empower non-binary voices and co-create knowledge that informs service design (Israel et al., 2005).

Most importantly, future studies must ensure methodological inclusivity by explicitly incorporating non-binary identity categories into data collection tools. Researchers should avoid conflating non-binary individuals with MSM or transgender populations unless data are disaggregated and identities clearly defined (Bauer et al., 2017). The absence of clear gender identity measures in most existing studies documented in this review—represents both a data gap and an advocacy opportunity.

Finally, future research should overcome language and publication bias by including studies published in French, Portuguese, and indigenous African languages. Incorporating multilingual data sources will allow for a more representative understanding of the health realities facing Black non-binary individuals across the subcontinent (Glänzel et al., 2016; Grépin et al., 2017).

## 5 Conclusion

This systematic review and meta-analysis provided critical insights into the health disparities faced by black non-binary individuals in sub-Saharan Africa. The key finding is that this population is at significantly more risk of mental health problems, in particular depression and anxiety, with pooled odds ratios of 2.85 and 2.47, respectively. Additionally, physical health outcomes, such as Sexually Transmitted Infections (STIs), were found to be more prevalent, with black non-binary individuals exhibiting a threefold higher likelihood of STIs compared to the general population. A major contributing factor to these poor health outcomes was the limited access to healthcare services, particularly gender-affirming care, with 75% of participants reporting barriers, such as discrimination and mistreatment. These results underscore the intersection of health inequities, stigma and systemic discrimination faced by this marginalized group in the sub-Saharan African context.

### 5.1 Policy and health intervention recommendations

The findings of this study underscore the urgent need for comprehensive policy reforms and health interventions to address the systemic inequities faced by Black non-binary individuals in sub-Saharan Africa. Central to these reforms is the integration of gender-affirming care into national public healthcare systems. Gender-affirming services should be embedded within routine health service delivery to ensure that non-binary individuals can access respectful, non-discriminatory, and culturally competent care that recognizes their specific needs (Reisner et al., 2016). Without institutionalizing such care, healthcare systems risk perpetuating exclusion and health disparities.

Equally critical is the development and implementation of targeted training programmes for healthcare providers. These should address implicit biases, increase awareness of gender diversity, and enhance clinical competencies in delivering affirming and inclusive care to non-binary populations (McGregor et al., 2019). Culturally responsive training will empower providers to engage with non-binary individuals in ways that respect their identities and foster trust.

Given the high prevalence of psychological distress within this population, there is also a pressing need to strengthen mental health support systems. Mental health services should be accessible, comprehensive, and specifically designed to respond to the unique stressors and trauma experienced by non-binary individuals within stigmatizing social environments (Mongelli et al., 2019). Such services should be integrated into primary healthcare to promote early intervention and reduce barriers to care.

In addition to clinical reforms, public health campaigns play a pivotal role in reshaping societal attitudes. Awareness initiatives should aim to educate communities about the existence, rights, and dignity of non-binary individuals, thereby reducing stigma and promoting social inclusion. These campaigns must be supported by robust anti-discrimination laws and policies that are actively enforced in both healthcare and broader social contexts (World Health Organization, 2009).

Furthermore, governments and health agencies should invest in systematic data collection and research funding to better understand the health needs of non-binary populations. Current data gaps limit the development of evidence-based interventions. Prioritizing research that includes disaggregated data on gender identity will enhance the effectiveness and relevance of health policies and services (Glänzel et al., 2016).

Together, these interventions will help establish a more inclusive, equitable, and responsive healthcare system one that is capable of safeguarding the health and human rights of Black non-binary individuals across sub-Saharan Africa.

### 5.2 Concluding remarks

Black non-binary individuals in sub-Saharan Africa continue to experience profound health inequities, shaped by systemic discrimination and social marginalization. The findings of this study demonstrate the urgent need for healthcare reform, along with an opportunity to build more inclusive and more affirming healthcare environments. By addressing the stigma and discrimination that disproportionately affect this population, policymakers and health practitioners can reduce health disparities and promote the well-being of black non-binary individuals. This should form the basis for future research, which uses longitudinal studies, and the crafting of interventions that will address the root causes of health inequities. Through concerted efforts, there is potential to achieve significant progress in promoting health equity for black non-binary individuals in the region.

## Data Availability

All data analysed during the course of this study were extracted from publicly available published literature, which is comprehensively cited throughout the manuscript. Any additional information related to the data extraction matrix, coding protocols, or statistical summaries can be made available by the corresponding author upon reasonable request.
